# Efficacy of a Combined Antiplatelet Therapy Is Not Affected by a Simultaneous Binding of Cangrelor and PSB 0777 to Albumin

**DOI:** 10.3389/fphar.2021.638257

**Published:** 2021-03-11

**Authors:** Joanna Wzorek, Radosław Bednarek, Cezary Watala, Magdalena Boncler

**Affiliations:** ^1^ Department of Haemostasis and Haemostatic Disorders, Medical University of Lodz, Lodz, Poland; ^2^ Department of Cytobiology and Proteomics, Medical University of Lodz, Lodz, Poland

**Keywords:** human serum albumin, cangrelor, adenosine analogue, fluorescence spectroscopy, surface plasmon resonance, molecular modeling, dual antiplatelet therapy

## Abstract

Concurrent administration of two drugs may complicate the management of acute coronary syndromes: competitive drug displacement diminishes drug binding and alters drug pharmacodynamics. We investigated the interaction of two antiplatelet compounds (PSB 0777 and cangrelor) with human serum albumin (HSA) to determine whether they compete with one another for the binding to albumin. Both examined compounds have been earlier claimed to bind to HSA (PSB 0777) or plasma proteins (cangrelor). Fluorescence spectroscopy, surface plasmon resonance spectroscopy and molecular modeling indicated that PSB 0777 and cangrelor interacted with HSA with moderate affinity (K_D_∼10^−5^ M). The binding of cangrelor to HSA involved primarily hydrophobic interactions, while the interaction of PSB 0777 with HSA was driven by hydrophobic and electrostatic forces. It was found that PSB 0777 and cangrelor do not share the same binding site on the protein. Our findings highlight the importance of albumin in the transport of PSB 0777 and cangrelor and suggest that the antiplatelet activity of the examined compounds used in combination is not affected by competition-induced changes in drug binding to HSA.

## Introduction

The binding to plasma proteins is a complex and dynamic process which can profoundly influence the pharmacokinetics and pharmacodynamics of a drug. Such binding is explained by the free drug theory, according to which only unbound drugs can cross cell membranes and exert pharmacologic effects. As a consequence, drugs with high affinity for plasma proteins often demonstrate a relatively slow distribution and elimination and they require higher doses to achieve therapeutic effects *in vivo* (Curry, 2009; Bohnert and Gan, 2013).

Human serum albumin (HSA) is the major plasma protein, accounting for 60% of all plasma proteins, and one which plays a fundamental role in the transport of drugs, metabolites and endogenous ligands. Also, albumin holds great promise as a nanocarrier system for targeted drug delivery, in particular for the transport of anti-cancer drugs (Lamichhane and Lee, 2020; Mokaberi et al., 2020; Sharifi-Rad et al., 2020). Structurally, it is a non-glycosylated single polypeptide that folds into a heart shaped structure containing about 67% of *α*-helices, but no *ß*-sheet. The molecule of HSA consists of three homologous domains (I–III), each divided into two subdomains (A and B) that are stabilized by 17 disulfide bridges (Rabbani and Ahn, 2019). The capacity of HSA to bind a large variety of drugs originates from the presence of two binding sites, namely, Sudlow site I and Sudlow site II, located—respectively, in the subdomains IIA and IIIA (Sudlow et al., 1976). Many ligands have been shown to bind to the both sites, although with different affinities. Sudlow site I favors the binding of anionic heterocyclic compounds, while small aromatic carboxylic acids bind preferably to Sudlow site II (Lambrinidis et al., 2015). Two commonly known site markers of Sudlow site I and II are warfarin and ibuprofen, and these are applied in the competitive studies for identification of drug binding sites on HSA (Fanali et al., 2012; Graciani and Ximenes, 2013; Poor et al., 2015).

Significant changes in drug binding to HSA and drug action *in vivo* may be elicited by the presence of other drugs. Co-administration of two drugs may lead to diminished plasma drug binding, being a result of either competitive displacement from the same binding site or allosteric displacement following microenvironmental changes at the binding site (Yamasaki et al., 2013). For example, Setoguchi et al. report that co-administration of diclofenac and nabumetone for arthritic pain treatment resulted in a marked increase in the concentration of free diclofenac and improvement of pharmacological effect due to displacement from HSA of the former by the latter (Setoguchi et al., 2013). Similar alterations may potentially occur in dual antiplatelet therapy, consisting of the combination of two platelet inhibitors; this is the mainstay of the pharmacological treatment of patients with acute coronary syndrome, particularly those undergoing percutaneous coronary intervention (Valgimigli et al., 2018). If that was the case, then the overall efficacy of antiplatelet drugs would be a result of drug-target interactions preceded by a direct competition between the drugs for the binding sites on albumin.

Recently, one of the adenosine receptor agonists, PSB 0777, was found to effectively inhibit platelet function and potentiate the antiplatelet effects of the P2Y_12_ receptor inhibitor, cangrelor (Boncler et al., 2018). Furthermore, HSA was revealed to interact with PSB 0777 and significantly reduce its antiplatelet activity (Wzorek et al., 2020). It is very likely that cangrelor also binds to HSA, as far as it highly binds to plasma proteins (97‒98%) (Keating, 2015). However, the influence of plasma proteins on the antiplatelet action of cangrelor has not been established so far. Likewise, the binding sites for PSB 0777 and cangrelor on HSA have not been identified.

The aim of this study was to analyze the interactions of human serum albumin with two antiplatelet compounds, *viz.* PSB 0777 and cangrelor, and to assess the involvement of the primary drug binding sites on HSA, Sudlow sites I and II, in these interactions. The results collected under the same conditions allowed a reliable determination of the extent to which both compounds bind to HSA and whether they were able to compete with each other for HSA binding. Our findings demonstrate that both antiplatelet compounds interacted with HSA, and that the dissociation constants of the formed complexes (PSB 0777-HSA and cangrelor-HSA) were in the order of 10^−5^ M. However, it appeared that they did not share the same binding site on the protein. Since PSB 0777 and cangrelor do not compete for the binding with HSA, it could be concluded that effectiveness of PSB 0777 and cangrelor, when used in combination, is not associated with the changes in formation of complexes between the examined compounds and HSA.

## Materials and Methods

### Chemicals

PSB 0777 (ammonium salt) was purchased from Tocris Bioscience (Bristol, United Kingdom) and cangrelor (sodium salt, AR-C69931MX) was from Cayman Chemical (Ann Arbor, MI, United States). Human serum albumin (HSA, purity 96–99%), warfarin sodium salt, ibuprofen sodium salt, and adenosine 5′-diphosphate (ADP, purity ≥95%), prostaglandin E_1_ (PGE_1_) were obtained from Sigma (St. Louis, MO, United States). Monoclonal antibodies anti-human CD61/PerCP (Cat# 347408, RRID: AB_2811174), CD62/PE (Cat# 348107, RRID: AB_2184974), mouse IgG1/PE isotype control (Cat# 340013, RRID: AB_399997) and Cellfix were obtained from Becton Dickinson (San Diego, CA, United States). Biacore sensor chips (CM5), Biacore amine coupling kit and BIAmaintenance kit were from GE Healthcare (Little Chalfont, United Kingdom). The BCA Protein Assay Kit was purchased from Thermo Scientific (Waltham, MA, United States). Phosphate buffered saline (PBS) was obtained from Biomed-Lublin (Lublin, Poland). All other chemicals, unless otherwise stated, were purchased from Avantor Performance Materials (Gliwice, Poland). All reagents were of analytical grade. Water used for solution preparation and glassware washing was passed through an Easy Pure UF water purification system (Thermolyne Barnstead, IA, United States).

### Experimental Design

Before the binding efficiency of cangrelor and PSB 0777 to albumin could be compared, it was necessary to demonstrate that cangrelor could bind to human serum albumin. The binding of cangrelor to HSA was evaluated by surface plasmon resonance (SPR). In addition, the effect of plasma and HSA on the antiplatelet action of cangrelor was investigated in biological samples: platelet-rich plasma and suspensions of isolated platelets. Fluorescence spectroscopy was the primary method of investigation used to study the interactions of cangrelor and PSB 0777 with HSA. This method was applied to achieve three different goals. The first was to learn to which extent the antiplatelet agents were able to interact with HSA under the same experimental conditions. For this purpose, the fluorescence spectra of HSA were recorded in the presence of cangrelor or PSB 0777. Second, to elucidate the quenching mechanism of HSA induced by antiplatelet compounds, a thermodynamic analysis of cangrelor and PSB 0777 binding to HSA was conducted. Third, to explore the contribution of two well-defined drug binding sites on albumin, namely, Sudlow site I and II, in the interactions of cangrelor and PSB 0777 with HSA; to achieve this, we studied the competitive binding of antiplatelet compounds (cangrelor and PSB 0777) and warfarin (probe for Sudlow site I) or ibuprofen (probe for Sudlow site II) to albumin. In addition, the displacement of PSB 0777 from albumin by cangrelor was examined. Finally, the binding site of cangrelor and PSB 0777 on albumin was predicted by molecular modeling.

### Surface Plasmon Resonance Measurements

The binding of cangrelor to immobilized human serum albumin was assessed by surface plasmon resonance spectroscopy using the Biacore X system (Biacore AG, Uppsala, Sweden). The experiments were carried out on a dual channel CM5 sensor chip in PBS buffer, pH 7.4, containing 0.005% surfactant P20 as previously described (Wzorek et al., 2020). Briefly, 100 µg of HSA was dissolved in 1 ml of 10 mM sodium acetate buffer, pH 4.0, and immobilized on the flow cell 1 of the sensor chip surface. No protein was immobilized on the flow cell 2, which was used as a control. Sequential injections of cangrelor solution at increasing concentrations (2, 5, 10 µM) were performed in a running buffer flushed on the sensor chip surface at the flow rate of 30 µl min^−1^; an injection volume of 50 µL was administered for 100 s, followed by a 100 s dissociation time and subsequent regeneration. The curves from three independent experiments were analyzed by global curve fitting of the entire data set using the Langmuir binding model with the BIAevaluation 4.1.1 software (Biacore AG, Uppsala, Sweden).

### Subjects and Blood Sample Collection

Human blood was obtained from consecutively recruited healthy volunteers (simple random non-stratified sampling): four men and seven women; mean age 25 ± 5 years. All individuals provided their written informed consent to participate in the study. The exclusion criteria were the same as described previously (Wzorek et al., 2020). The study was approved by the Human Studies Committee of the Medical University of Lodz (Poland) and was conducted in accordance with the guidelines established by the Declaration of Helsinki. Whole blood was collected into polypropylene tubes with a blood-to-acid citrate dextrose solution (ACD) ratio of 6:1 (vol:vol).

### Platelet Preparation

Human platelet-rich plasma (PRP) was prepared by centrifuging of anticoagulated whole blood at 190 x g for 12 min at 37°C. Platelet-poor plasma (PPP) was prepared by centrifuging the whole blood fraction left after removal of the PRP at 2500 x g for 12 min at 37°C. Platelet count in PRP was measured with automated haematology analyzer Sysmex XS-800i™ (Sysmex, Kobe, Japan) and adjusted to 1 × 10^8^ cells per ml.

Suspensions of isolated platelets were obtained from PRP separated from anticoagulated whole blood containing 50 ng/ml PGE_1_. PRP was centrifuged at 800 x g for 15 min at 37°C to obtain a platelet pellet. Platelet pellet was immediately suspended in the Tyrode buffer (134 mM NaCl, 12 mM NaHCO_3_, 2.9 mM KCl, 0.34 mM Na_2_HPO_4_, 1 mM MgCl_2_, 10 mM HEPES, 5 mM glucose, pH 7.4) and diluted to a count 1 × 10^8^ per ml, as described above.

### Analysis of Platelet Activation

The effect of plasma or HSA on inhibition of platelet activation by cangrelor was assessed by two-colour flow cytometry with the use of anti-CD61/PerCP (for platelet gating) and anti-CD62/PE (for detection of activated platelets). The antiplatelet activity of cangrelor was evaluated in PRP and suspensions of isolated platelets, when the effect of plasma was examined, or in isolated platelets containing 4% HSA, when the effect of albumin was studied. Cell response (a fraction of activated platelets) was measured after preincubation of samples with 17 nM (IC_50_) cangrelor (Boncler et al., 2018) dissolved in PBS buffer (3 min, 37°C) followed by a 5-min stimulation with 10 μM ADP. The activated platelets were stained with antibodies prior to fixation as described previously (Wzorek et al., 2020). A total of 10,000 events were acquired in each experiment using a FACSCanto II flow cytometer and surface expression of CD62P (P-selectin) in platelets was analysed using FACS/Diva v. 6.0 software (Becton-Dickinson, Franklin Lakes, NJ, United States).

### Fluorescence Spectroscopy

The fluorescence measurements for albumin-drug complexes were performed using a LS50B spectrofluorometer (Perkin Elmer, Waltham, MA, United States) equipped with 1.0 cm quartz cell. The fluorescence spectra for HSA solution (2.5 µM in 0.1 M phosphate buffer, pH 7.4) were recorded in the range of wavelength from 250 to 500 nm, with the excitation at 280 nm and the emission set at 350 nm. The slit widths for excitation and emission beams were set to 5 and 10 nm, respectively. The Perkin-Elmer FL WinLab software v. 4.00.03 was used to collect the measured data. An appropriate buffer blank spectrum was subtracted from the measured spectra for fluorescence background correction. The optical density of HSA solution was below 0.1 and within the albumin concentration range of 0‐2.5 µM we observed a linear relationship between protein concentration and fluorescence intensity (*p* < 0.0001, R^2^ = 0.995). In consequence, the albumin fluorescence was not corrected for the inner filter effect. All measurements, except a thermodynamic analysis of the binding of antiplatelet compounds to HSA were performed at 25°C after 30 s incubation of a sample with the examined compound. Stock and working solutions of all compounds used in the experiments were prepared in PBS buffer, pH 7.4. The protein concentration was determined by BCA method (Smith et al., 1985).

#### Fluorescence Quenching of Albumin by Antiplatelet Compounds

To study the mechanism by which cangrelor and PSB 0777 quench the fluorescence of HSA, the fluorescence spectra for HSA were recorded in the presence of increasing concentrations of antiplatelet compounds. Cangrelor and PSB 0777 were applied in two concentration ranges: 0‒5 µM (with 0.5 µM intervals, 1:2 protein to drug molar ratio) and 0‒38.5 µM (with 4 µM intervals, 1:15 protein to drug molar ratio). The parameters useful for predicting the mechanism of albumin quenching by the antiplatelet compounds (quenching constants: K_sv_ and K_q_) were determined as described previously (Magdum et al., 2017). To obtain the binding parameters: dissociation constant (K_D_) and the number of binding sites per protein molecule (n), the experimental data were fit to a saturation binding curve using nonlinear regression analysis. Briefly, quenching constants (Stern-Volmer quenching constant, quenching rate constant) were determined using the Stern–Volmer equation Eq. 1:

$${\text{F}}_{\text{0}}/\text{F}=1+{\text{K}}_{\text{q}}\tau_{\text{0}}\left[\text{Q}\right]=1+{\text{K}}_{\text{SV}}\left[\text{Q}\right],$$ (1)

where F_0_ and F denote the fluorescence intensities of the protein before and after the addition of the quencher; K_q_ is the quenching constant, τ_0_ is the lifetime of the fluorophore in the absence of quencher, K_SV_ is the Stern-Volmer quenching constant (equal to the dynamic quenching constant), and Q is the concentration of the quencher. For a static quenching interaction, the values of K_D_ and n were calculated using the Eq. 2:

$$1-\left(\text{F}/{\text{F}}_{\text{0}}\right)=\text{n}\left[\text{Q}\right]/\left(\left[{\text{K}}_{\text{D}}\right]+\left[\text{Q}\right]\right)$$ (2)

Thermodynamic parameters, such as the free energy (ΔG^θ^), enthalpy (ΔH^θ^) and entropy (ΔS^θ^), are important to describe the forces acting between antiplatelet compound and HSA. To obtain such information, fluorescence spectra of HSA were recorded at three different temperatures: 292, 302, and 312 K in the presence of cangrelor or PSB 0777. The antiplatelet compounds were used in the concentration range of 0‒38.5 µM (2.5, 14.5, 26.5, and 38.5 µM). According to the binding constants obtained at three temperatures, the values of ΔH^θ^ and ΔS^θ^ were determined from van’t Hoff plots by fitting ln K_b_ vs. 1/T using the van’t Hoff equation (Eq. 3), where R is gas constant and T is temperature (in K):

$$\text{ln}\;{\text{K}}_{\text{b}}=-\Delta {\text{H}}^{\theta }/\text{RT}+\Delta {\text{S}}^{\theta }/\text{R}$$ (3)

The free energy change was estimated from the following relationship Eq. 4:

$$\Delta {\text{G}}^{\theta }=\Delta {\text{H}}^{\theta }-\text{T}\Delta {\text{S}}^{\theta }$$ (4)

#### Synchronous Fluorescence

To monitor changes in the polarity around the tyrosine and tryptophan residues of HSA evoked by the examined compounds, the synchronous fluorescence spectra of HSA (Δλ 15 nm and Δλ 60 nm) were recorded in the presence of increasing concentrations of PSB 0777 and cangrelor (0‒38.5 μM with 4 μM intervals).

#### Fluorescence Measurements in the Presence of Competitors

To identify the binding sites of antiplatelet compounds on HSA, fluorescence measurements were carried out with the use of warfarin and ibuprofen, which are established site markers for Sudlow site I and II, respectively (Ni et al., 2006). The study was performed by titration of HSA with the antiplatelet compound (cangrelor or PSB 0777 at the concentration range of 0‒38.5 µM with 4 µM intervals, 1:15 protein to drug molar ratio) in the presence of warfarin or ibuprofen. In accordance with previous reports, the binding site marker and albumin were used in equimolar concentrations (2.5 µM) (Zhang and Wang, 2011; Chaves et al., 2018; Manjushree and Revanasiddappa, 2019). Displacement experiments were preceded by a determination of the dissociation constant and the number of binding sites for warfarin and ibuprofen forming a complex with HSA. These parameters were obtained by measuring the fluorescence intensity of HSA in the presence of site marker used at the concentration range of 0–5 µM (with 0.5 µM intervals, 1:2 protein to drug molar ratio).

Furthermore, to determine whether one antiplatelet compound could be displaced from HSA by the other, the binding site marker experiments were repeated, i.e. by titration of HSA with PSB 0777 (0‒38.5 µM) in the presence of 2.5 µM cangrelor, instead of a marker.

### Molecular Docking

Molecular docking of the antiplatelet compounds (cangrelor and PSB 0777) to human serum albumin was performed with the AutoDock 4.2.6 software (The Scripps Research Institute, La Jolla, CA, United States) (Morris et al., 2009) using the Lamarckian genetic algorithm (GA) for a flexible ligand and a rigid receptor docking.

The 3D structures of PSB 0777 (ID: 90488932) and cangrelor (ID: 8029718) were downloaded from PubChem (http://pubchem.ncbi.nlm.nih.gov) and ChemSpider (http://www.chemspider.com) respectively and converted to PDB format using Open Babel 2.4.1 software (O’boyle et al., 2011). The ligand molecules were prepared for the proper use of AutoDock semi-empirical free energy force field as follows: hydrogen atoms were added, Gasteiger charges were computed, non-polar hydrogens were merged, aromatic carbons were detected, the torsional degrees of freedom were set up, and the rotatable (flexible) and rigid bonds were identified. Cangrelor and PSB 0777 were docked to the two X-ray high resolution crystal structures of HSA: 2BXD and 2BXG (Ghuman et al., 2005). The crystal structures of HSA were obtained from the Research Collaboratory for Structural Bioinformatics Protein Data Bank (RCSB PDB; http://www.rcsb.org). The 2BXD structure is complexed with warfarin (R-enantiomer) and contains a major binding pocket for drugs (Sudlow site I) located in the core of subdomain IIA. This binding site comprises six helices of the subdomain IIA (residues 196‒297) and a loop-helix feature of the subdomain IB (residues 148–154). The 2BXG structure is complexed with ibuprofen and contains the second drug binding site (Sudlow site II), which consists of six helices located in subdomain IIIA (residues 384–497) (Sudlow et al., 1976; Ghuman et al., 2005). The water molecules and both the co-crystalized ligands used, warfarin and ibuprofen, were removed from each PDB structure, respectively, 2BXD and 2BXG, using UCSF Chimera 1.13.1 software (Pettersen et al., 2004). Likewise, two structures of HSA with either Sudlow site I and Sudlow site II in a ligand-accepted conformation, but actually without any ligand bound, were obtained. Using AutoDock software, all missing hydrogen atoms were added at appropriate geometry within both HSA structures, Gasteiger charges were computed and non-polar hydrogens were merged. The active sites in HSA molecule were defined by amino acid residues that are important for drug binding, namely Sudlow site I (TYR150, LYS195, LYS199, PHE211, TRP214, ALA215, ARG218, ARG222, LEU238, HIS242, ARG257. and ILE264 in the 2BXD structure) and Sudlow site II (ARG348, GLU383, ARG410, TYR411, LYS414, VAL433, GLU450, ARG485, and SER489 in the 2BXD structure) (Al-Harthi et al., 2019). In both HSA structures the possible histidine tautomers with the proton on either Nδ1 or Nε2 atoms were sampled. HIS242 residue that is found in Sudlow site I was sampled as Nδ1 protonated tautomer in 2BXD HSA structure. The coordinates for the initial compound sites were calculated by the UCSF Chimera 1.13.1 software using the “Define Centroid” tool. For 2BXD structure, we calculated the coordinates for complexed warfarin, whereas for 2BXG structure, the coordinates for complexed ibuprofen were calculated to verify, whether the tested compounds interact with either Sudlow site I or Sudlow site II. The calculated coordinates were as follows (x, y, and z): 3.736, (−9.600) and 5.600 for the 2BXD structure and 5.048, (−4.464) and (−15.042) for the 2BXG structure. Accordingly, the grid size for the 2BXD structure was set to 60 × 60 × 60 xyz points with the grid spacing of 0.375 Å. For the 2BXG structure, the grid size was set to 60 × 90 × 50 xyz points with the grid spacing of 0.375 Å. Both grids were centered at the coordinates calculated for the initial compound sites. Docking was performed using Lamarckian genetic algorithm (GA) with the following parameters used: the maximum number of energy evolutions: 5,000,000; GA population size: 150; the maximum number of generations: 27,000; the number of top individuals to survive to the next generation: 1; and the number of GA runs: 60. The HSA-ligand complex with the lowest values of the Gibbs free binding energy (ΔG_b_) and the inhibition constant (K_i_) was taken as the most optimal conformation.

### Statistical Analysis

The results are expressed as mean ± SD or mean with the 95% confidence intervals. Data were collected in at least three independent experiments. The normality of the data distribution was verified with the Shapiro-Wilk test. Normally distributed raw data was analysed with the paired *t*-test, unpaired *t*-test, one-way ANOVA or repeated measures ANOVA with the *post hoc* Dunnett’s multiple comparisons test (if F was significant). Variables that did not meet the assumptions for parametric tests were assessed with the Mann-Whitney U-test, Wilcoxon’s signed ranks test or Friedman’s test with the post-hoc Dunn’s multiple comparisons test. Linear regression was used to assess association between protein concentration and fluorescence intensity. Saturation binding curves were fit by nonlinear regression (one-site binding model) to determine affinity of the examined compounds for albumin. The statistical analysis was performed using the following software packages: Statistica v.13.3, GraphPad Prism v.5, and StatsDirect v.2.8.0.

## Results

### SPR Analysis of the Binding of Cangrelor to HSA

**FIGURE 1:**
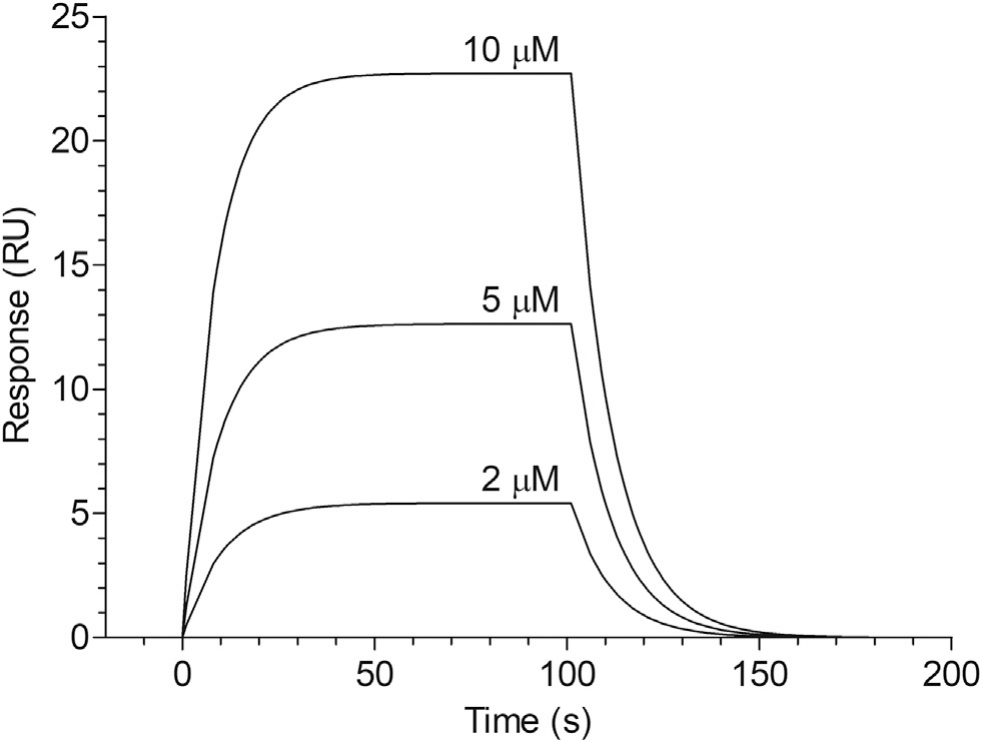
Surface plasmon resonance sensorgram of cangrelor binding to HSA. HSA was immobilized to a dextran-coated gold surface. Injection of analyte (cangrelor at the concentration of 2, 5, or 10 µM) produced a signal change that was directly proportional to the mass of bound drug molecules, expressed in resonance units (RU). Further details are given in “Materials and Methods” section.

The dose-dependent binding of cangrelor to HSA is shown in Figure 1. The estimates of the kinetic rate constants obtained from the Langmuir model were as follows: k_a_ = 2.82 ± 1.40 × 10^3^ M^−1^ s^−1^ and k_d_ = 5.73 ± 3.23 × 10^−2^ s^−1^ (*n* = 3). Moreover, the equilibrium association and dissociation constants (K_A_ and K_D_) obtained from the binding rate constants were: K_A_ = 6.12 ± 4.68 × 10^4^ M^−1^ and K_D_ = 2.37 ± 1.55 × 10^−5^ M. The goodness of fit, as indicated by the averaged chi-square value (χ^2^) for set of experiments was 2.14 ± 0.47.

### Influence of Plasma and HSA on Inhibition of Platelet Activation by Cangrelor

**FIGURE 2:**
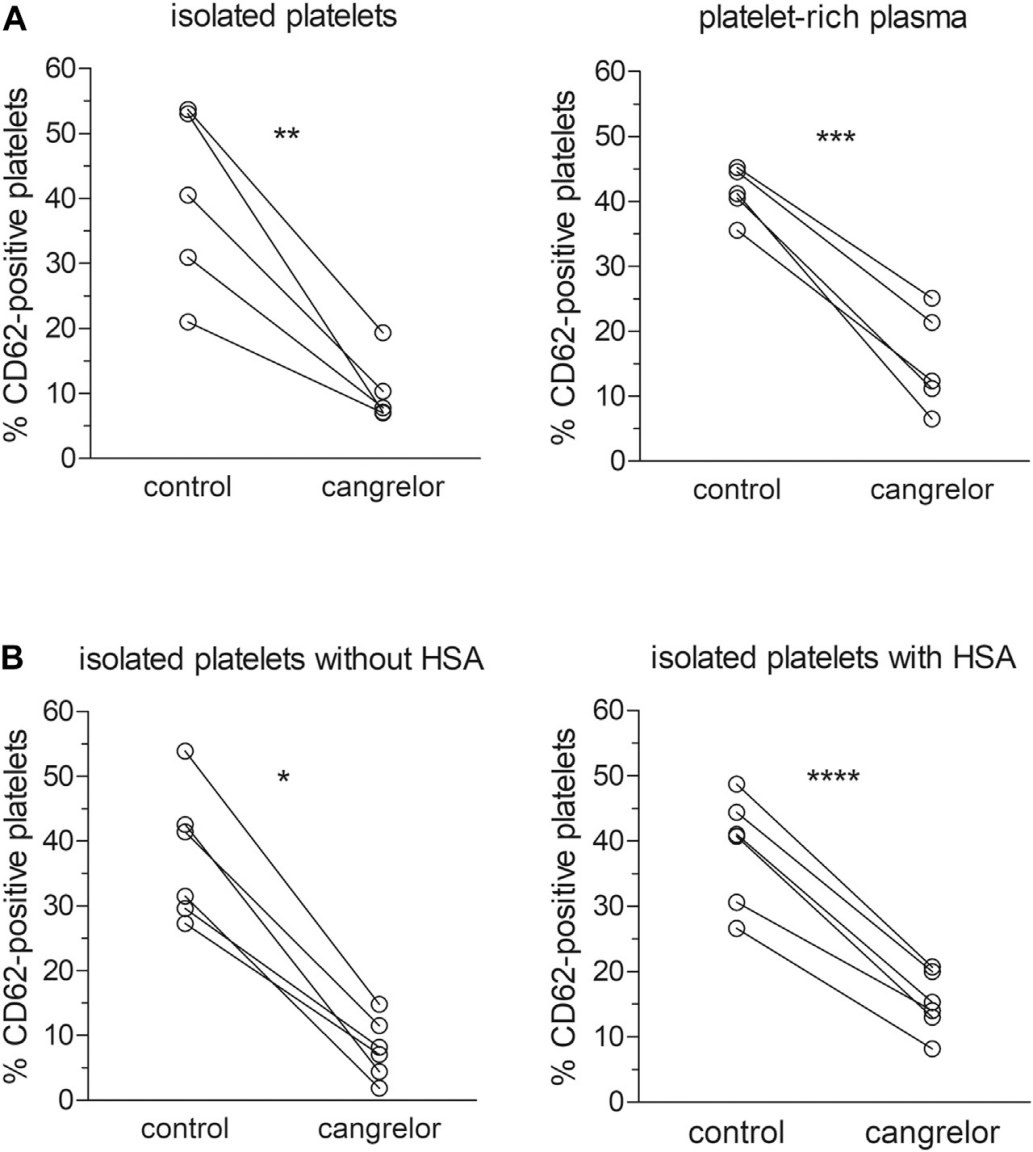
Inhibition of platelet activation by cangrelor. The antiplatelet activity of cangrelor was evaluated in the presence of plasma proteins (**A**, *n* = 5) or 4% HSA (**B**, *n* = 6) compared to control. PRP or platelet suspensions were pre-incubated with 17 nM cangrelor (3 min, 37°C) and platelet activation was measured by flow cytometry in response to 10 μM ADP. Platelet activation was analyzed on the basis of platelet P-selectin expression (fraction of CD62P-positive cells). Statistically significant differences in platelet response to ADP between the groups were estimated with the paired Student t test or Wilcoxon test. Isolated platelets: µ_cangrelor_ ≠ µ_control_ (*p* < 0.01), platelet-rich plasma: µ_cangrelor_ ≠ µ_control_ (*p* < 0.001), isolated platelets without HSA: µ_cangrelor_ ≠ µ_control_ (*p* < 0.05), isolated platelets with HSA: µ_cangrelor_ ≠ µ_control_ (*p* < 0.0001).

To assess the influence of plasma constituents on the antiplatelet activity of cangrelor, the platelet response of PRP to cangrelor was compared with that of suspensions of isolated platelets. Platelet activation in response to 10 μM ADP was increased from 7.4 ± 4.4 to 41.4 ± 3.8% in PRP and from 8.9 ± 6.0 to 39.9 ± 14.2% in suspensions of isolated platelets (*n* = 5). No statistically significant differences in spontaneous and ADP-induced platelet activation between PRP and isolated platelets were observed. Cangrelor at a concentration of 17 nM decreased ADP-dependent platelet activation by 77% (*p* < 0.001) in PRP and by 95% (*p* < 0.01) in isolated platelets (Figure 2A). The average inhibition of platelet activation was significantly lower in PRP compared to isolated platelets (1.2-fold difference, *p* < 0.05).

The effect of albumin on the anti-platelet action of cangrelor was tested in suspensions of isolated platelets with 4% HSA and those without (controls). After stimulation with 10 μM ADP, the fraction of CD62P-positive platelets rose from 6.1 ± 4.0 to 37.7 ± 10.1% in controls and from 7.1 ± 4.7 and 38.7 ± 8.4% in those treated with 4% HSA (*n* = 6). Cangrelor used at 17 nM inhibited ADP-induced platelet activation by 74% (*p* < 0.0001) in suspensions of isolated platelets with 4% HSA and by 97% (*p* < 0.05) in control platelets (Figure 2B). The mean inhibition of platelet activation evoked by cangrelor was significantly lower (1.3-fold, *p* < 0.01) in platelet suspensions with HSA compared to controls. All inhibition values were calculated after subtraction of basal response.

### Fluorescence Quenching Study

#### Interactions of Cangrelor and PSB 0777 with HSA

Fluorescence quenching of HSA by cangrelor and PSB 0777 was initially investigated at low (0‒5 µM) and high (0‒38.5 µM) concentration ranges. The basal fluorescence intensity of HSA measured at 350 nm was in the range of 394–537 relative fluorescence units (RFU) (*n* = 17).

In the concentration range of 0–5 μM, both cangrelor and PSB 0777 gradually decreased the fluorescence intensity of HSA with increasing drug concentration. However, HSA fluorescence was rather moderately diminished by the examined compounds (by 25% in the presence of PSB 0777 (*n* = 5) and by 40% in the presence of cangrelor (*n* = 6)); the difference in fluorescence quenching of albumin between the compounds was significant at *p* < 0.001).The binding for both cangrelor and PSB 0777 used in the concentration range of 0–5 μM was weak, and therefore, we were not able to reliably calculate the binding parameters (K_D_ and n).

**FIGURE 3:**
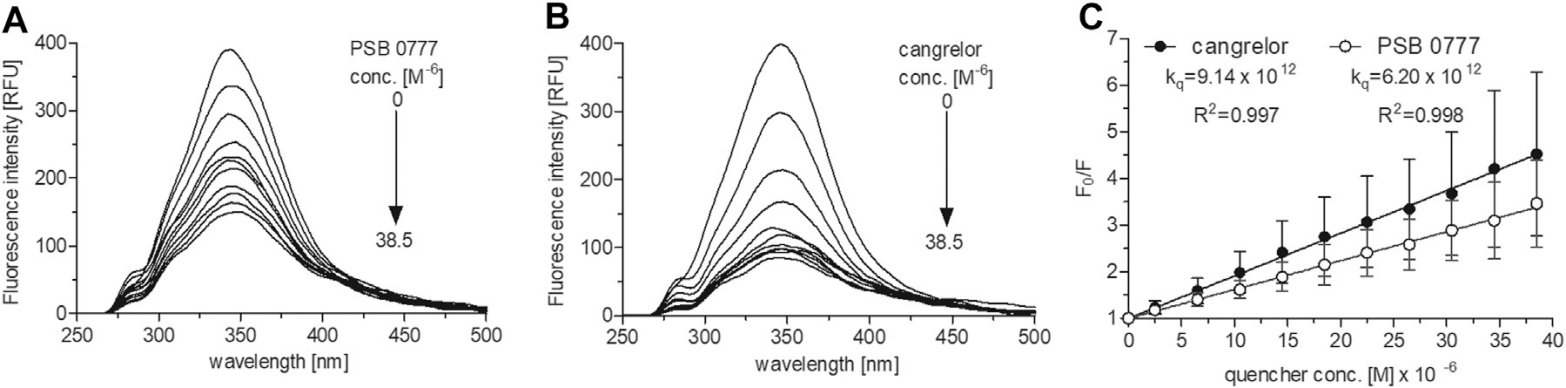
Fluorescence emission spectra and Stern-Volmer plots of HSA fluorescence quenching by PSB 0777 and cangrelor. The spectra of HSA (2.5 µM) were recorded at RT in PBS, pH 7.4, in the presence of increasing concentration of PSB 0777 **(A)** or cangrelor **(B)**. The Stern–Volmer curves **(C)** were obtained by plotting the relative fluorescence intensity as a function of quencher concentration to obtain Stern–Volmer quenching constants. Data represent mean ± SD of three independent experiments. Further details are given in “Materials and Methods” section.

At 0‒38.5 µM both agents effectively decreased HSA fluorescence, with substantial changes being seen at the lowest concentrations (*p* < 0.001 for all drug concentrations) (Figures 3A,B). At the highest drug concentration (38.5 µM), HSA fluorescence was diminished by 75% in the presence of cangrelor (*n* = 3) and by 70% by PSB 0777 (*n* = 3), although the changes were not statistically different between the compounds. The Stern-Volmer plots for the interaction of HSA with cangrelor and PSB 0777 at 0‒38.5 µM exhibited a linear trend (Figure 3C). The quenching rate constants did not significantly differ between cangrelor and PSB 0777 (Figure 3C). Moreover, the values of K_q_ obtained for both compounds were at least 300 times higher than 2 × 10^10^ M^−1^ s^−1^ (the maximum value for scattering collision quenching constant). Such observations indicate that the mechanism of HSA fluorescence quenching by cangrelor and PSB 0777 resulted from the formation of complexes between the examined antiplatelet compounds and HSA (a mechanism of static quenching was predominant).

Furthermore, there was no significant difference in the dissociation constants between cangrelor and PSB 0777 (1.2-fold difference, NS). The numbers of binding sites in HSA for cangrelor and PSB 0777 were close to 1 (Table 1). Based on the above observations, further fluorescence spectroscopic experiments were carried out only at the higher concentration range of cangrelor and PSB 0777.

**TABLE 1:** Binding parameters for the interactions of PSB 0777 and cangrelor with HSA.

Quencher	Binding constants		Number of binding sites	R^2^
	K_D_ × 10^−5^ M	K_A_ × 10^4^ M^−1^	n	
PSB 0777 (0‒38.5 M^−6^)	1.36 [−0.93 to 3.65]	7.36 [−0.11 to 2.74]	0.83 [−0.22 to 1.87]	0.933
Cangrelor (0‒38.5 M^−6^)	1.09 [−1.02 to 3.20]	9.17 [−9.82 to 3.13]	0.99 [−0.29 to 2.27]	0.850

The binding constants and number of binding sites for the interaction of PSB 0777 and cangrelor with human serum albumin are presented as best-fit values calculated using nonlinear regression analysis. Data show mean values with the 95% confidence intervals (*n* = 3). K_A_ = 1/K_D_.

**FIGURE 4:**
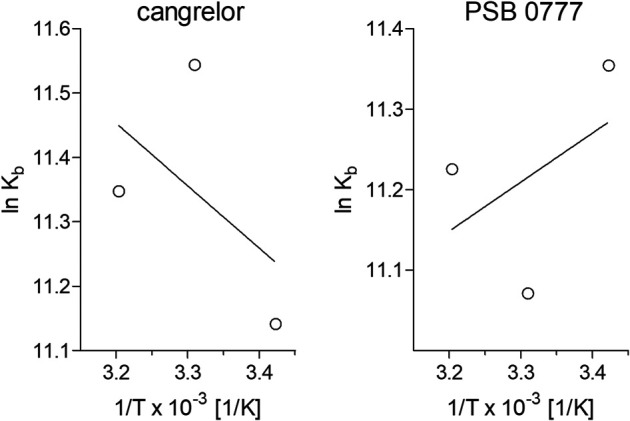
Van’t Hoff plots of the temperature dependence of the equilibrium constant for the complexes of HSA with cangrelor and PSB 0777. To determine thermodynamic parameters of reaction, the binding of cangrelor and PSB 0777 to HSA was monitored at three different temperatures: 292, 302, and 312 K. More details are given in “Materials and Methods” section.

Determination of binding constants for cangrelor and PSB 0777 at various temperatures, followed by the calculation of thermodynamic parameters, allowed us to describe the intermolecular forces acting between the examined compounds and HSA. The K_D_ and K_A_ values for cangrelor and PSB 0777 measured at various temperatures slightly differed, however the differences remained statistically insignificant in all the cases (Table 2). According to the binding constants measured at 292, 302 and 312 K, the thermodynamic parameters were determined from a linear van’t Hoff plot (Figure 4) and presented in Table 2. The reaction between cangrelor or PSB 0777 and HSA was spontaneous because the values of the free energy changes (ΔG^θ^) calculated for both compounds were negative at all temperatures. Other thermodynamic parameters (the values of enthalpy and entropy) indicated the involvement of hydrophobic interactions in the formation of the cangrelor-HSA complexes (ΔH^θ^ > 0, ΔS^θ^ > 0). The interaction between PSB 0777 and HSA was most likely driven by hydrophobic forces and, to some extent, electrostatic interactions (ΔH^θ^ < 0, ΔS^θ^ > 0).

**TABLE 2:** Thermodynamic parameters for the binding of PSB 0777 and cangrelor to HSA.

Quencher	T [K]	K_D_ × 10^−5^ [M]	K_A_ × 10^4^ [M^−1^]	ΔS^θ^ [J/mol * K]	ΔH^θ^ [kJ/mol]	ΔG^θ^ [kJ/mol]
PSB 0777	292	1.17 [−0.34 to 2.69]	8.53 [−0.29 to 3.72]	76.49	−5.06	−27.41
	302	1.56 [−1.91 to 5.02]	6.43 [−5.24 to 1.99]			−28.17
	312	1.33 [0.11‒2.56]	7.50 [−0.90 to 3.91]			−28.94
Cangrelor	292	1.45 [0.94‒1.96]	6.90 [0.11‒5.11]	121.01	8.06	−27.29
	302	0.97 [0.51‒1.44]	10.31 [0.20‒6.97]			−28.50
	312	1.18 [0.52‒1.84]	8.47 [0.19‒5.45]			−29.71

Thermodynamic parameters were calculated based on the K_A_ values, which were determined in the experiments of HSA quenching by cangrelor or PSB 0777 (0‒38.5 µM) at the temperatures of 292, 302, and 312 K. Binding constants are expressed as mean and 95% CI (*n* = 3). K_A_ = 1/K_D_.

The synchronous fluorescence spectra provided us information about the molecular environment in the vicinity of the HSA molecules in the presence of the examined compounds. In general, upon the gradual addition of PSB 0777 and cangrelor to HSA solution the fluorescence intensity of HSA decreased; no spectacular red shift was observed in the emission maxima of the tyrosine and tryptophan residues. In the presence of PSB 0777, the peak of emission shifted to a longer wavelength; the mean shift was by 0.5 nm at Δλ 15 nm for tyrosine and by 0.8 nm at Δλ 60 nm for tryptophan. Upon the addition of cangrelor, a more pronounced difference in the red shift was obtained at Δλ 15 and 60 nm: mean difference was 0.3 nm at Δλ 15 nm and by 2.1 nm at Δλ 60 nm. The results indicate that PSB 0777 and cangrelor slightly increased the polarity around the tyrosine and tryptophan residues, however the most visible change was induced by cangrelor in the microenvironment around the TRP214 residue, which is located in the subdomain IIA of albumin. The representative synchronous fluorescence spectra of interaction of PSB 0777 and cangrelor with HSA are presented in Supplementary Figure S1.

#### Cangrelor and PSB 0777 Interactions with HSA in the Presence of Binding Site Markers—Competitive Experiments

**FIGURE 5:**
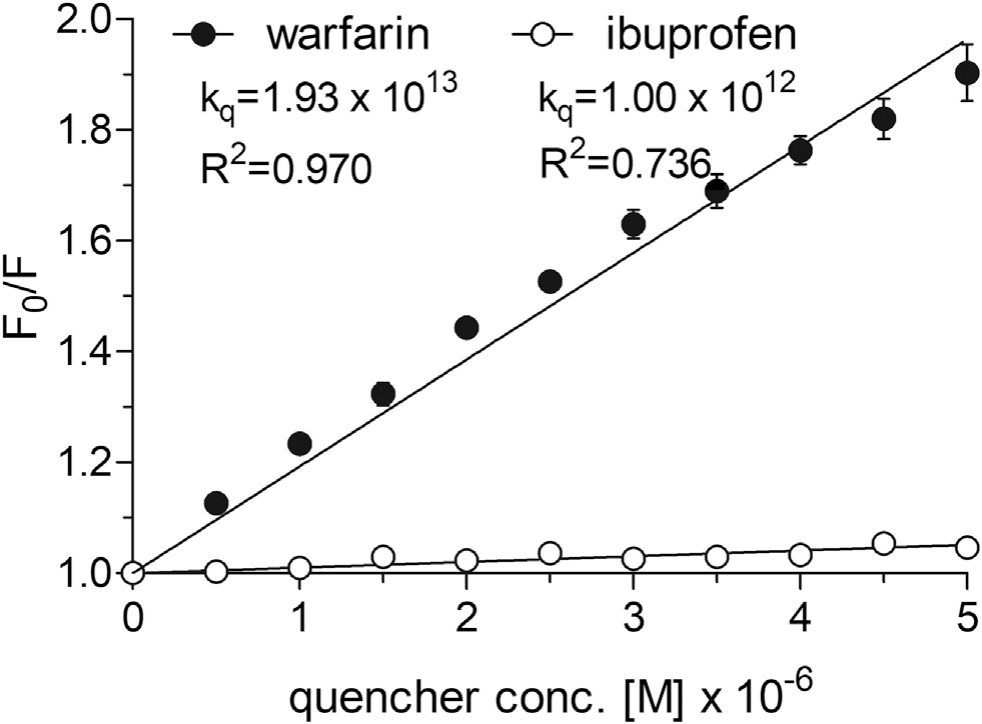
Stern-Volmer plots and quenching constants for the interactions of warfarin and ibuprofen with HSA. The Stern–Volmer curves were obtained by plotting the relative fluorescence intensity as a function of quencher concentration to obtain Stern–Volmer quenching constants. Data represent mean ± SD of three independent experiments. Further details are given in “Materials and Methods” section.

To determine the dissociation constant and the number of binding sites for warfarin and ibuprofen being in a complex with albumin, we measured the fluorescence intensity of HSA in the presence of warfarin and ibuprofen over the concentration range of 0–5 µM. As shown in Figure 5, warfarin effectively decreased the fluorescence intensity of HSA (a decrease was from 11% at 0.5 µM, *p* < 0.0001, to 47% at 5 μM, *p* < 0.0001) and the effect was dose-dependent (*p* < 0.0001). Ibuprofen also quenched the intrinsic fluorescence of HSA in a dose-dependent manner (*p* < 0.0001), however its effect did not exceed 5% (at 4.5‒5.0 µM, *p* < 0.0001). Consequently, the K_sv_ and K_q_ values obtained for ibuprofen were nearly 20-fold lower than those recorded for warfarin (Figure 5). Since ibuprofen was not found to be an effective quencher for albumin, it was excluded from further analysis. The K_D_ value and the number of binding site for the warfarin-HSA complex were respectively: 3.38 [1.66‒5.09] × 10^−6 ^M and 0.86 [0.41‒1.31]. Here, it is worth noting that the K_D_ value determined for warfarin was approximately of one order of magnitude lower from that obtained for cangrelor or PSB 0777; this indicated that warfarin could be used as a marker for Sudlow site I. In the presence of warfarin or cangrelor acting as competitors for PSB 0777, the dissociation constants for PSB 0777-HSA complex were slightly but insignificantly higher than in the controls (1.4-fold higher for warfarin and 1.2-fold higher for cangrelor, NS). In contrast, a significant increase in the dissociation constant for cangrelor-HSA complex was observed in the presence of warfarin compared to control (2-fold increase, *p* < 0.015) (Table 3). Based on these observations, it could be inferred that cangrelor and warfarin (but not PSB 0777) bind to the same site on HSA.

**TABLE 3:** Dissociation constants and the number of binding sites for the interactions of PSB 0777 and cangrelor with HSA in the presence of competitor.

Quencher	Competitor	Without competitor		With competitor	
		K_D_ × 10^−5^ M	n	K_D_ × 10^−5^ M	n
PSB 0777	Warfarin	1.08 [0.64‒1.51]	0.69 [0.51‒0.88]	1.52 [0.22‒2.82]	0.59 [0.19‒0.99]
	Cangrelor	1.00 [0.27‒1.73]	0.61 [0.32‒0.89]	1.28 [0.57‒1.99]	0.65 [0.39‒0.92]
Cangrelor	Warfarin	0.83 [0.28‒1.37]	1.10 [0.67‒1.52]	1.68 [−0.42 to 3.79]	1.39 [−0.07 to 2.85]
	PSB 0777	ND	ND	ND	ND

ND: not determined. K_D_ values are expressed as mean and 95% CI (*n* = 5–6). The dissociation constants and the number of binding sites for the drug-HSA complexes were investigated in the absence and presence of a competitor: warfarin (for PSB 0777 and cangrelor) or cangrelor (for PSB 0777). Differences between the groups were analyzed using paired *t* test.

### Docking of Cangrelor and PSB 0777 to Sudlow Sites I and II of HSA

**FIGURE 6:**
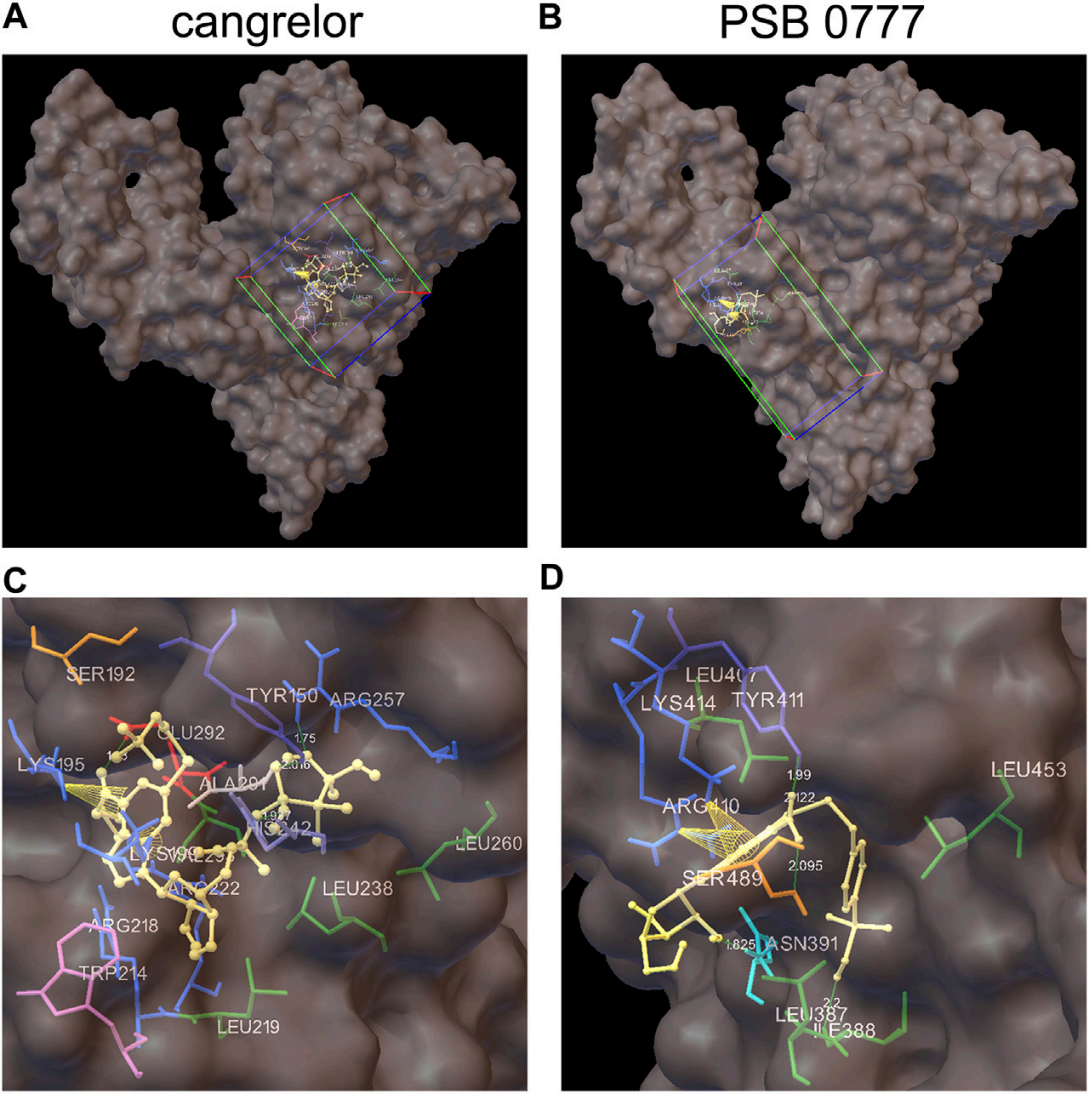
Docking of cangrelor and PSB 0777 within the binding pockets of the HSA molecule. Cangrelor and PSB 0777 were docked to the two different crystal structures of HSA: 2BXD (albumin complexed with warfarin) and 2BXG (albumin complexed with ibuprofen). **(A, C)**: cangrelor complexed with albumin (2BXD) within Sudlow site I at different magnification. **(B, D)**: PSB 0777 complexed with albumin (2BXG) within Sudlow site II at different magnification. Cube (on the left) and cuboid (on the right) indicate the location and size of the grid.

The spectroscopic results described above indicate that the main binding site for cangrelor in HSA is Sudlow site I in subdomain IIA. It is also likely that Sudlow site II, being the second major drug binding site of albumin, could be engaged in the interaction of PSB 0777 with HSA. Hence, these two hydrophobic pockets were chosen for performing computational experiments that aimed to provide a more detailed information on the interaction between the examined compounds and albumin. Molecular docking results indicated that the binding of cangrelor with Sudlow site I of HSA and PSB 0777 to Sudlow site II of HSA is possible. Figure 6 shows PSB 0777 and cangrelor docked into the hydrophobic pockets of HSA, Sudlow site I and II with the use of the 2BXD and 2BXG crystal structures. Docking results indicate that these interactions have an exothermic nature, in which van der Waals forces, hydrogen bonding and desolvation play the main role (Table 4).

**TABLE 4:** Energy values obtained during docking analysis of PSB 0777 and cangrelor as a ligand and human serum albumin as a target protein.

Ligand (drug binding site, PDB ID of HSA structure)	PSB 0777 (Sudlow site II, 2BXG)	cangrelor (Sudlow site I, 2BXD)
Estimated free energy of binding (kcal/mol) [= (1) + (2) + (3) − (4)]	−5.86	−6.24
Estimated inhibition constant, K_i_ (µM) at temperature = 298.15 K	50.29	26.73
(1) final intermolecular energy (kcal/mol)	−9.44	−12.80
vdW + Hbond + desolv energy	−9.49	−12.18
Electrostatic energy	+0.04	−0.62
(2) final total internal energy	−3.94	−4.32
(3) Torsional free energy	+3.58	+6.56
(4) Unbound system’s energy [=(2)]	−3.94	−4.32

The energy values were evaluated by the molecular docking software Autodock 4.2.6. Estimated free energy of binding corresponds to Gibbs free energy, defined as: ΔG_b_ = −RT lnK, where R is ideal gas constant, T is the room temperature (298.15 K), and K is equilibrium constant. From the mathematical point of view, K = K_D_ = K_i_.

PSB 0777 was found to be in close contact with the nine amino acid residues in subdomain IIIA of HSA: LEU387, ILE388, ASN391, LEU407, ARG410, TYR411, LYS414, LEU453, and SER489. Residues ILE388, ASN391, TYR411, and SER489 participated in hydrogen bonding with PSB 0777 (in all, five hydrogen bonds were detected in the PSB 0777-HSA complex), whereas residues LYS414 and ARG410 were involved in π-cation interactions with the adenine ring of PSB 0777.

As regards cangrelor, the following amino acid residues of albumin were detected to be involved in the complex formation with HSA: TYR150, SER192, and LYS195 from subdomain IB and LYS199, TRP214, ARG218, LEU219, ARG222, LEU238, HIS242, ARG257, LEU260, ALA291, GLU292, VAL293 from subdomain IIA. Hydrogen bonds were formed between TYR150, ARG257, HIS242, GLU292 and the heterocyclic adenine ring of cangrelor. HIS242 was assigned as the Nδ1 protonated tautomer and served as the H-bond acceptor in the complex formation. Moreover, intramolecular π–cation interactions between the heterocyclic adenine ring of cangrelor and albumin residues LYS195 and LYS199 also occurred.

## Discussion

Concurrent administration of two drugs, as used in dual antiplatelet therapy, may lead to diminished protein-drug binding in plasma and tissues, resulting from competitive displacement of one drug by another from a carrier (Yamasaki et al., 2013). Such pharmacokinetic changes can positively affect the concentration of the free (unbound) drug and overall effect of antiplatelet therapy. In the present study, we hypothesized that two compounds with antiplatelet activity, PSB 0777 (adenosine receptor agonist, an adenosine derivative) and cangrelor (P2Y_12_ receptor inhibitor, an adenosine triphosphate analogue), compete with each other for binding on human serum albumin, thus providing the additional, non-receptor mechanism underlying the effectiveness of a combined antiplatelet therapy. Significantly, our results indicate that: 1) both cangrelor and PSB 0777 bind to HSA with moderate affinities and their antiplatelet actions are significantly decreased in the presence of plasma or HSA, 2) cangrelor binds to Sudlow site I in subdomain IIA, whereas the binding site of PSB 0777 on HSA is most likely located within subdomain IIIA in Sudlow site II, 3) cangrelor and PSB 0777 do not compete for the same binding site on HSA, as far as their binding sites on the protein are different. Based on these findings it can be concluded that effectiveness of cangrelor and PSB 0777 is not associated with competition-induced changes in drug binding to HSA.

Considering the issue of displacement of one antiplatelet agent by another from HSA, it was critical to demonstrate that PSB 0777 and cangrelor are transported by the same carrier. Recently, we found that PSB 0777 significantly inhibits platelet aggregation and enhances the antiplatelet effects of cangrelor, but its antiplatelet activity strongly depends on the presence of plasma or HSA. Moreover, using SPR technology, the binding of PSB 0777 to HSA was detected with the affinity of 8.06 × 10^−5^ M (Boncler et al., 2018; Wzorek et al., 2020). As regards cangrelor, it has been reported that it is strongly bound to plasma proteins (Keating, 2015), however, there is neither direct nor indirect evidence of cangrelor binding to albumin. Therefore, in the present study, the influence of plasma or HSA on the antiplatelet action of cangrelor using platelet-rich plasma and suspensions of isolated platelets containing HSA was examined. It also investigated the interaction of cangrelor with purified HSA by SPR.

Incubation of control samples with cangrelor resulted in nearly complete inhibition of platelet activation in response to ADP. This inhibition was reduced in the presence of plasma or HSA, thus highlighting the importance of plasma constituents, in particular albumin, as modulators of cangrelor effectiveness in inhibiting of platelet function (Figure 2). Furthermore, SPR measurements indicated that cangrelor binds to HSA. Cangrelor showed moderate affinity for HSA (K_D_ = 2.37 × 10^−5^ M), although the value of equilibrium dissociation constant was over three times lower than that obtained earlier for PSB 0777, indicating that cangrelor has a higher affinity for albumin compared to PSB 0777 (Wzorek et al., 2020). It could be noticed that cangrelor was used at 10-fold lower concentration than PSB 0777, which resulted from a difference in their IC_50_ values required for effective inhibition of platelet function in whole blood: the apparent cell response to antagonist was achieved at nanomolar concentration of cangrelor and a micromolar concentration of PSB 0777 (Boncler et al., 2018). The difference in concentration ranges, however, should not influence the results, because, in principal, the values of the rate constants and the equilibrium constants calculated from the rate constants are independent of the concentrations of both analyte and ligand (Sabapathy et al., 2020).

To investigate the interactions of PSB 0777 and cangrelor with HSA, two non-separative, moderate-throughput methods were used: SPR and fluorescence spectroscopy (Huber and Mueller, 2006; Vuignier et al., 2010). SPR is successfully used to rank drug molecules of weak, medium, and strong affinity to albumin and α_1_-acid glycoprotein. Spectroscopic assays are better suited to high-affinity systems, but looking at the literature, they are often used in the studies on HSA interactions with drugs (Alanazi and Abdelhameed, 2016; Rabbani et al., 2017; Rub et al., 2017) that, in general, are of moderate affinity (Otagiri, 2005). The use of the latter method allowed us to examine the interactions of antiplatelet compounds with HSA in solution and provided the insight into the drug binding mechanism.

Studies of the quenching of intrinsic fluorescence of albumin are carried out at equimolar/close-to-equimolar concentrations of quencher and albumin (Dufour and Dangles, 2005; Manjushree and Revanasiddappa, 2019) or at higher molar concentrations of quencher compared to albumin (Graciani and Ximenes, 2013; Meng et al., 2015; Goszczynski et al., 2017), depending on how strong the fluorescence quenching of albumin is. In the preliminary experiments, when the antiplatelet compounds were used at close to an equimolar ratio with albumin, they moderately quenched the HSA fluorescence and the binding parameters could not be reliably estimated by nonlinear regression. Therefore, further analysis was conducted at higher molar ratio of drug to protein (15:1). As expected, PSB 0777 and cangrelor, used at 0‒38.5 µM, induced a strong HSA quenching (the fluorescence of albumin was quenched up to 75%) and statistically significant changes in HSA fluorescence were already seen at the lowest concentration of the examined compounds. The formation of complexes between PSB 0777 or cangrelor and HSA in a static quenching model was deduced from the values of quenching constants (k_q_), which were much greater than the maximum scatter collision quenching constant. This observation indicated that the mechanism of HSA fluorescence quenching is not caused by dynamic collision, where a direct contact of the fluorophore and quencher is not required (Yeggoni et al., 2015; Alanazi and Abdelhameed, 2016). Moreover, PSB 0777 and cangrelor were found to bind to a single site on HSA. The mean K_D_ for cangrelor was shown to be 1.2-fold lower than that obtained for PSB 0777 but both values remained of the same order of magnitude (∼10^−5^ M) and indicated a moderate affinity of the examined compounds for albumin. Many ligands bind to HSA with a moderate binding affinity (Otagiri, 2005); hence, the obtained binding constants for PSB 0777 and cangrelor seem to be high enough to ensure effective drug distribution (Bohnert and Gan, 2013). The values of thermodynamic parameters and the van’t Hoff plots were indicative for character of reactions, which were: exothermic in the case of PSB 0777 and endothermic for cangrelor. In the cangrelor-HSA system, the interaction was mainly driven by hydrophobic forces, as confirmed by the positive ΔS^θ^ and ΔH^θ^ values. The interaction between PSB 0777 and HSA was accompanied by an increase in entropy and decrease in enthalpy, so it is likely that hydrophobic forces and electrostatic interactions are involved in the formation of PSB 0777-HSA complexes. A similar binding mechanism as PSB 0777-HSA has been demonstrated for other adenosine analogues (N^6^-(2-hydroxyethyl)-adenosine, 8-bromoadenosine, cordycepin) and adenosine itself (Cui et al., 2007; Cui et al., 2008; Cui et al., 2009; Meng et al., 2015).

To sum up, data obtained by SPR and fluorescence spectroscopy are consistent, providing evidence for the binding of PSB 0777 and cangrelor to HSA. This allowed us to further investigate the interactions of the examined compounds with HSA. In particular, we were interested whether PSB 0777 and cangrelor bind to Sudlow site I or Sudlow site II, since the binding of ligands with HSA occurs mainly in these regions (Sudlow et al., 1976; Ghuman et al., 2005; Al-Harthi et al., 2019). For this purpose, we decided to use warfarin and ibuprofen, which are specific ligands for Sudlow site I and II, respectively (Ni et al., 2006). Surprisingly, in control experiments with selected displacers, only warfarin was capable to bind with HSA and therefore could serve as a competitor. The value of association constant for warfarin bound to HSA determined in our study (K_A_ = 2.96 × 10^5^ M^−1^) was consistent with values reported earlier (K_A_ is within the range of 10^4^–10^5^ M^−1^) (Yamasaki et al., 1996; Watanabe et al., 2001; Dufour and Dangles, 2005) and, importantly, was of one order of magnitude higher than association constants determined for PSB 0777 and cangrelor (Table 1). Interestingly, the binding constant of ibuprofen for HSA has been reported to be even higher from that for warfarin but it is when ibuprofen is used in the racemic form (K_A_ is within the range of 10^5^–10^6^ M^−1^) (Dufour and Dangles, 2005). Considering this, it could be inferred that ibuprofen preparation, which was applied in our laboratory and exhibited a negligible binding to HSA (Figure 5), was not a racemic mixture but consisted of a single enantiomer. Unfortunately, we cannot compare our results of HSA quenching by ibuprofen with the other competitive studies, because this kind of data is not collected or shown in those reports. Instead, we can find out from earlier publications what the binding constants are for the complex of drug-HSA, in the absence and presence of competitor. In our study, when warfarin was used as competitor, concurrent use of warfarin and cangrelor, led to a significant increase in the dissociation constant compared to control (cangrelor alone). This finding suggests that warfarin effectively displaces cangrelor from HSA, and hence that Sudlow site I is a binding site for cangrelor. Contrary to this, PSB 0777 was not displaced by warfarin, indicating that the binding site of PSB 0777 could be located in Sudlow site II. This is supported by additional experiments of displacement of PSB 0777 by cangrelor, which indirectly confirmed that PSB 0777 and cangrelor do not share the same binding site on albumin. Nevertheless, there is some possibility that both cangrelor and PSB 0777 bind to Sudlow site I. There is a persuasive argument in favour of this. Namely, it has been shown that Sudlow site I in the subdomain IIA forms a relatively large hydrophobic cavity (more voluminous than Sudlow site II) (Curry, 2009), able to bind more than one compound, without interfering with each other (Watanabe et al., 2001; Rimac et al., 2017). Moreover, according to the concept of Yamasaki et al., Sudlow site I may consist of at least three regions, partially overlapping, where three ligands could be bound (Yamasaki et al., 1996). Intriguingly, adenosine and 8-bromoadenosine have been successfully docked to the site I of human serum albumin, known as the warfarin binding site (Cui et al., 2008; Cui et al., 2009).

The competitive study has been combined with computational method ‒ molecular docking, to predict molecular interactions between PSB 0777 or cangrelor and HSA. Therefore, the antiplatelet compounds were docked into two hydrophobic pockets of HSA: PSB 0777 in the region containing Sudlow site II, and cangrelor in the region encompassing Sudlow site I. The results obtained by docking of PSB 0777 and cangrelor to HSA were in agreement with our *in vitro* observations. The only difference between the *in silico* method and the *in vitro* experiments could be found in the binding mode of PSB 0777 to HSA as electrostatic interactions were shown to play a minor role in the formation of PSB 0777-HSA complex in the mathematical model describing drug-HSA interaction (Table 4). As expected, PSB 0777 formed a complex with HSA via interactions with several amino acid residues located in subdomain IIIA, whereas cangrelor interacted with some residues of subdomain IIA and IB. The complexes of HSA with PSB 0777 and cangrelor were stabilized by hydrogen bonding interactions and π-cation interactions. Furthermore, the complexes were formed spontaneously, as indicated by negative values of Gibbs free energy. Consistent with the *in vitro* experiments is the finding showing that cangrelor has a lower free energy than PSB 0777, thus forming more stable complex with albumin. In the light of our findings we can conclude that albumin is important carrier for cangrelor and PSB 777 and the albumin-dependent bio-distribution of cangrelor seems not to be disturbed by the albumin-dependent bio-distribution of PSB 0777 in the course of the dual antiplatelet therapy.

In conclusion, quantitative data on the interactions between drug and carrier protein contribute to a better understanding the mechanisms of drug transport, its toxicity and action. Our results provide an insight into the role of interactions of platelet inhibitors with plasma proteins in shaping their effectiveness in dual antiplatelet therapy. To verify whether concurrent administration of two antiplatelet compounds may lead to diminished protein-drug binding in plasma and tissues, resulting from competitive displacement of one drug by another from a carrier, we studied the interactions of HSA with platelet P2Y_12_ inhibitor, cangrelor, and adenosine analogue PSB 0777 possessing the antiplatelet activity. Our findings demonstrated that both compounds bind to HSA, but do not compete for the same binding site on HSA. Sudlow site I seems to be the major region of cangrelor binding, whereas PSB 0777 is likely to interact with Sudlow site II or with another, not yet recognized region in albumin. Binding of drugs to albumin is a complex process, so that when studying drug-HSA interactions we have to bear in mind that albumin contains some regions, such as Sudlow site I, carrying several binding sites (Watanabe et al., 2001). On the other hand, some drugs, like warfarin, can bind to HSA at different sites of molecule (Dufour and Dangles, 2005). Considering this, crystallographic studies should help precisely determine the primary binding sites for PSB 0777 and cangrelor in albumin. We believe that our new concept of studying non-receptor mechanism underlying the effectiveness of dual antiplatelet therapy, which combines many disciplines, such as medicine, pharmacy, biology, physics, chemistry, may be a very good starting point for further investigation.

## Data Availability

The original contributions presented in the study are included in the article/Supplementary Material, further inquiries can be directed to the corresponding author.
