# Supplementary figures and images for: Efficacy of a Combined Antiplatelet Therapy Is Not Affected by a Simultaneous Binding of Cangrelor and PSB 0777 to Albumin
Source: Front Pharmacol. 2021 Mar 11;12:638257. doi: 10.3389/fphar.2021.638257 (PMC7990796; doi:10.3389/fphar.2021.638257)

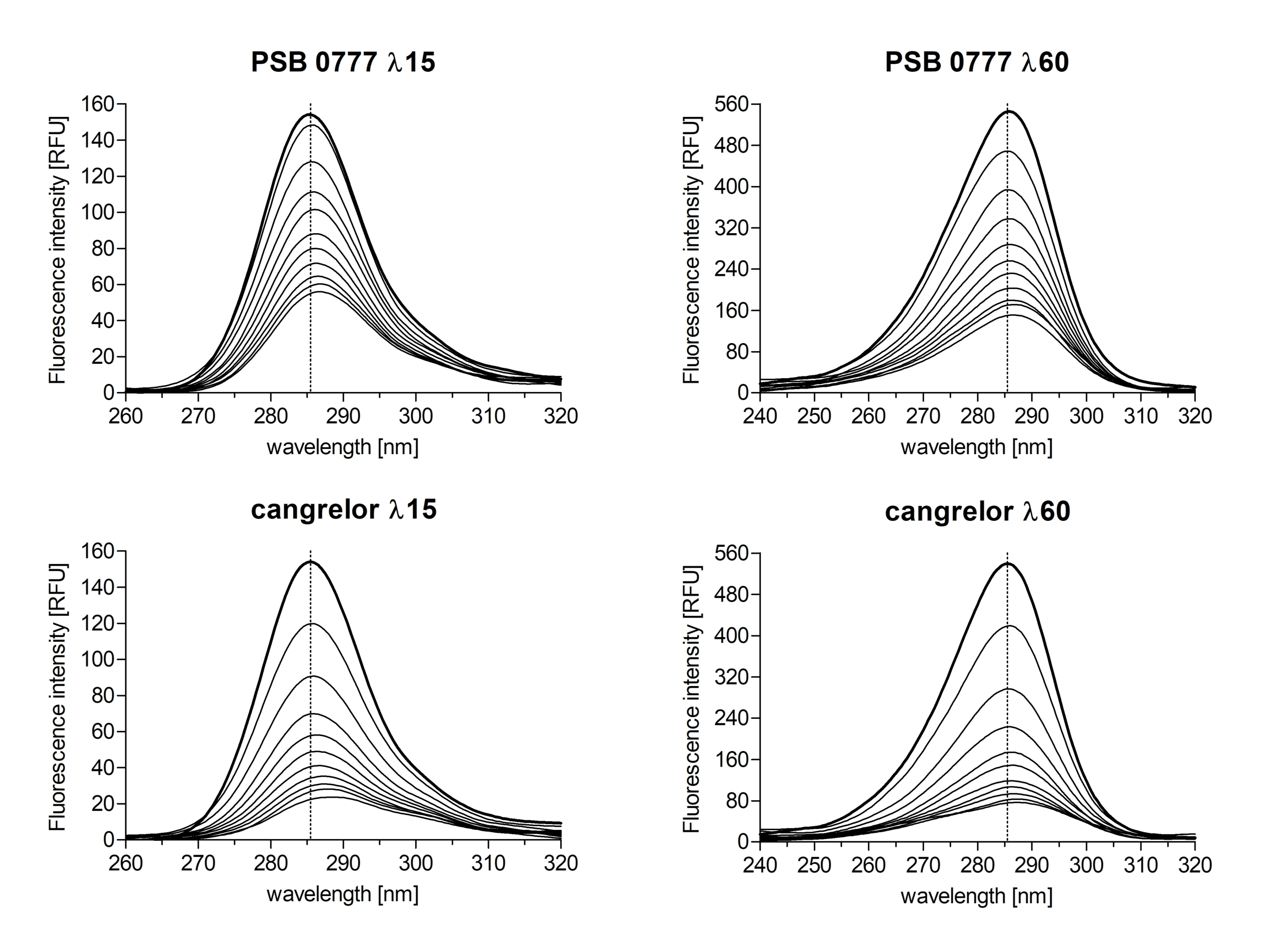

Supplement: Supplementary file 1 [file image1.tif]
